# Impact of changing classification systems on prevalence and frequency distribution of odontogenic tumors in tertiary care center of Nagpur

**DOI:** 10.1016/j.bjorl.2021.02.006

**Published:** 2021-03-07

**Authors:** Harpreet Kaur, Suchitra Gosavi, Vinay K. Hazarey, Vandana Gupta, Upendra Singh Bhadauria, Pooja Kherde

**Affiliations:** a All India Institute of Medical Sciences, Centre for Dental Education and Research, Division of Oral Pathology and Microbiology, New Delhi, India; b Government Dental College & Hospital, Department of Oral Pathology, Nagpur, India; c Datta Meghe Institute of Medical Sciences Deemed University, Nagpur, India; d All India Institute of Medical Sciences, Centre for Dental Education and Research, Division of Periodontics, New Delhi, India; e All India Institute of Medical Sciences, Centre for Dental Education and Research, National Oral Health Programme, New Delhi, India

**Keywords:** Odontogenic tumors, Changing classifications, WHO 2017 system, Frequency distribution, Demographics

## Abstract

**Introduction:**

The classification of odontogenic tumors has been revised from time to time in order to provide unified terminology. This reclassification had considerable impact on their prevalence and frequency distribution.

**Objectives:**

This study was aimed to emphasize impact of changing classification systems on prevalence and relative frequency of odontogenic tumors. The secondary objective was to analyze demographics of various histological types of odontogenic tumors in comparison to published literature. Review of Indian studies (1992–2020) elaborating frequency of odontogenic tumors is summarized in the end.

**Methods:**

This was a hospital-based retrospective study wherein case files of odontogenic tumors diagnosed from 1990 to 2019 period were retrieved. The classification system used originally at the time of diagnosis was retained and prevalence of odontogenic tumors in three different periods (1990–2004, 2005–2016 and 2017–2019) was compared. Further, prevalence, frequency distribution and demographics of all these tumors (1990–2019) were analyzed using latest World Health Organization 2017 classification.

**Results:**

A total of 345 odontogenic tumors was diagnosed as per World Health Organization 2017 system of classification from 1990 to 2019. 96.81% tumors were benign and 3.81% constituted malignant odontogenic tumors. However, there was marked increase in prevalence of odontogenic tumors in 2005–2016 (6.2%) period as compared to 1990–2004 (3.87%) and 2017–2019 (3.47%). Ameloblastoma remained the most common tumor in three different periods, whereas keratocystic odontogenic tumor became second commonest tumor in 2005–2016 as compared to odontoma in 1990–2004 and adenomatoid odontogenic tumor in 2017–2019.

**Conclusions:**

The continuous evolving systems of classification may partly be responsible for inconsistency in odontogenic tumors, with inclusion of keratocystic odontogenic tumor,and has marked impact on prevalence and frequency distribution of odontogenic tumors. The geographical variations in demographics of odontogenic tumors might reflect genetic and environment influence; however it requires elucidation by further studies.

## Introduction

Odontogenic tumors (OTs) are a histologically diverse group of lesions derived from the odontogenic apparatus.^1^ The literature is replete with studies on incidence, prevalence and epidemiology of OTs worldwide showing considerable variation in their distribution.^2^, ^3^ The difference in the frequency distribution of OTs is seen not only among different parts of the world,^2^, ^3^, ^4^, ^5^, ^6^, ^7^, ^8^, ^9^, ^10^, ^11^, ^12^, ^13^, ^14^, ^15^ but also within Indian regions.^1^, ^16^, ^17^, ^18^, ^19^, ^20^, ^21^, ^22^, ^23^, ^24^ Besides regional variations, uncertainties in the designation of some lesions as cysts or neoplasms might be the major confounders contributing to variability in prevalence and frequency distribution of OTs.

An initial attempt to categorize these diverse tumors under a unified umbrella of OTs was made by Broca (1869), following which different systems were published to define uniform diagnostic criteria. Finally, an extensive 5 year study led to the first consensus proposal and authoritative classification guide by the World Health Organization (WHO) in 1971 to emphasize standard terminology internationally, which was later modified in the second edition (1992).^25^, ^26^ With the advent of advanced ancillary techniques, an updated third edition was proposed by Philipsen and Reichart in 2005.^27^, ^28^ The most significant revisions of this edition were Reclassifications of odontogenic keratocyst (OKC) and calcifying odontogenic cyst (COC) as neoplasms. However, controversies persisted about existence, nomenclature, and diagnosis of various entities. After much debate, WHO put forth a fourth edition of simplified version of classification in 2017, wherein OKC and COC were relocated as cysts with inclusion of some new entities.^29^

Gaitán-Cepeda LA et al. (2010), Bianco et al. (2020), Jaeger F et al. (2016) and Servato JPS et al. (2013) discussed the impact of reconsideration of OKC as neoplasm on frequency distribution of OTs.^5^, ^30^, ^31^, ^32^, ^33^ However, to the best of our knowledge, to date none of the studies compared prevalence and frequency distribution of OTs in comparison of the previous classification schemes (WHO 1992 and 2005) to latest WHO 2017 classification.

Thus, this retrospective study was designed to analyze the impact of changing classification systems on the prevalence and relative frequency of OTs. Further, relative frequency and demographics of various histological types of odontogenic tumors (1990–2019) were studied using WHO 2017 classification in central India. The secondary objective was to compare our results with published Indian literature of OTs.

## Methods

The study was approved by the research ethics committee of the Government Dental College and Hospital, Nagpur (reference nº IEC/03/26). The inclusion criteria comprised of OTs reported over a period of 30 years (1990–2019) along with available information such as demographics, clinical site and extent of involvement and, where paraffin embedded blocks, hematoxylin and eosin stained slides were available were included, whereas patients with incomplete information about age, sex, site and extent; availability of reports in records but without slides or paraffin embedded blocks were excluded from study.

In this retrospective study, histopathological records of all reported OTs over a period of 30 years (1990–2019) were analyzed and pertinent details were obtained from individual medical records. Hematoxylin and eosin-stained sections were reviewed independently by three oral pathologists. The OTs reported before 2005 (1992–2004) were classified as per WHO 1992 system: after 2005 (2005–2016) by WHO 2005 and after 2017 (2017–2019) by WHO 2017 classification. The sites were divided into mandible and maxilla, and regions were divided into anterior, posterior, unilateral anterior to posterior and lesions crossing midline. The ramus and angle region were also included in mandible. The prevalence and frequency distribution in three different periods were analyzed. Further overall prevalence, relative frequency, and demographics of all reported OTs (1990–2019) was elucidated using WHO 2017 system. Furthermore, the published English language literature was searched for OTs in India from 1990 to 2020 (Table 1).

**Table 1:** Summary of OTs reported in various regions of India.

S.no		Kadashetti et al.^19^ (WHO 2005) (Maharashtra)		Nalabolu et al.^17^ (WHO 2005) (Andhra Pradesh)		Varkhede et al.^21^ (WHO 2005) (Maharashtra)		Ebenezer et al.^24^ (WHO 2005) (South chennai)		Deepthi et al.^16^ (WHO 2005) (South kerala)		Gill S et al.^18^ (WHO 2005)(Gujarat)		Sriram G et al.^1^ (WHO 1992) (Maharashtra)		Bhagwat et al.^22^ (WHO 2005) (Maharashtra)		Ahire MS et al.^20^ (WHO 2017) (Maharashtra)		Gupta B et al.^23^ (WHO 1992) (Chennai)	
		Cases	%	Cases	%	Cases	%	Cases	%	Cases	%	Cases	%	Cases	%	Cases	%	Cases	%	Cases	%
1	Benign tumors																				
1.1	(Benign epithelial tumors	91.00	89.21%	146	90.6%	103	85.83%	33	33.33%	244	80%	167	81%	192	91.86%	111	87.40%	164	65.6%	382	78.11%
1.1.1	Ameloblastoma	37.00	36.27	79	49.06	49	40.83	15	14.02	153	50.2	99	47.4	154	61.6	45	35.43	124	49.6	331	67.7
1.1.2	AOT	5.00	4.9	9	5.5	7	5.83	3	2.8	12	3.93	16	7.7	31	12.4	6	4.72	35	14	44	8.9
1.1.3	CEOT	1.00	0.98	3	1.8	1	0.83	2	1.87	3	0.98	3	1.4	6	2.4	3	2.36	4	1.6	4	0.82
1.1.4	SOT	–	–	2	1.2	1	0.83	0	0	2	0.66	–	–	1	0.4	1	0.79	1	0.4	3	0.61
1.1.5	KCOT/OKC	48.00	47.05	53	32.9	45	37.5	13	12.15	74	24.3	49	23.4	–	–	56	44.09	–	–	–	–
1.2	(Benign mixed epithelial and mesenchymal tumors	9.00	8.82 %	11	6.83%	16	13.33%	64	64.6%	50	16.39%	26	12.6%	36	17.2%	13	10.2%	49	19.6%	46	9.4%
1.2.1	Ameloblastic fibroma	1.00	0.98	1	0.6	1	0.83	1	0.93	1	0.33	2	1	2	0.8	3	1.2	–	–	6	1.23
1.2.2	Ameloblastic fibrodentinoma	–	–	–	–	–	–	1	0.93	–	–	–	–	–	–	–	–	–	–	–	–
1.2.3	Ameloblastic fibroodontoma	–	–	–	–	–	–	–	–	2	0.66	–	–	3	1.2	–	–	–	–	1	0.2
1.2.4	Odontoameloblastoma	–	–	–	–	–	–	0	0	–	–	–	–	0	0	–	–	–	–	–	–
1.2.5	Odontoma- complex, compound	7.00	6.86	10	6.2	14	11.67	60	56.07	40	13.1	11	5.3	15	6	9	7.09	48	19.2	38	7.8
1.2.6	Calcifying cystic odontogenic tumor (CCOT) or COC/CEOC	1.00	0.98	–	–	1	0.83	2	1.87	7	2.3	13	6.2	16	7.6	1	0.79	–	–	1	0.2
1.2.7	Clear cell odontogenic tumor	–	–	–	–	–	–	–	–	–	–	–	–	0	0	–	–	–	–	–	–
1.2.8	Dentinogenic ghost cell tumor	–	–	–	–	–	–	–	–	–	–	–	–	–	–	–	–	1	0.4	–	–
1.3	(Benign mesenchymal tumors, 23 cases, 6.66%)	2.00	1.96%	4	2.48%	1	0.83%	1	1.01%	8	2.62%	13	6.31%	28	13.39%	4	3.14%	17	6.8%	19	3.88%
1.3.1	Odontogenic fibroma	1.00	0.98	–	–	–	–	1	0.93	4	1.31	–	–	12	4.8	–	–	5	2	2	0.4
1.3.2	Odontogenic myxoma	–	–	4	2.4	–	–	–	–	2	0.66	7	3.3	15	6	2	1.57	6	2.4	13	2.66
1.3.3	Cementoblastoma	1.00	0.98	–	–	1	0.83	–	–	2	0.66	6	2.9	1	0.4	2	1.57	6	2.4	4	0.82
2	Malignant tumors, 11 cases, 3.19%	–	–	–	–	–	–	1	0.93	3				3		2		1		15	
2.1	Ameloblastic carcinoma	–	–	–	–	–	–	–	–	1	0.33	–	–	3	1.2	–	–	1	0.4	–	–
2.2	Primary intra-osseous carcinoma	–	–	–	–	–	–	–	–	2	0.66	–	–	–	–	1	0.79	–	–	15	3.07
2.3	Clear cell odontogenic carcinoma	–	–	–	–	–	–	–	–	–	–	–	–	–	–	–	–	–	–	–	–
2.4	Ghost cell odontogenic carcinoma	–	–	–	–	–	–	–	–	–	–	–	–	–	–	1	0.79	–	–	–	–
2.5	Ameloblastic fibrosarcoma	–	–	–	–	–	–	–	–	–	–	–	–	–	–	–	–	–	–	–	–
2.6	Odontogenic sarcoma	–	–	–	–	–	–	–	–	–	–	–	–	–	–	–	–	–	–	–	–
2.7	Odontogenic carcinosarcoma	–	–	–	–	–	–	–	–	–	–	–	–	–	–	–	–				
	Total OTs	102.00	100	161	100	120	100	99	100	305	100	206	100	209	100	127	100	250	100	489	100

AOT, adenomatoid odontogenic tumor; CEOT, calcifying epithelial odontogenic tumor (Pindborg’s tumor); SOT, squamous odontogenic tumor; KCOT, keratocystic odontogenic tumor; OKC, odontogenic keratocyst; CCOT, calcifying cystic odontogenic tumor; CEOC, calcifying epithelial odontogenic Cyst; COC, calcifying odontogenic cyst; DGCT, dentinogenic ghost cell odontogenic tumor; OTs, odontogenic tumors.

### Statistical analysis

The data collected was subjected to statistical analysis using Statistical Package for Social Sciences (SPSS, IBM version 20.0). The level of significance was fixed at 5% and p ≤ 0.05 was considered statistically significant. Kolmogorov–Smirnov test and Shapiro-Wilks test were employed to test the normality of data.

## Results

Prevalence of OTs in three periods of classification: the prevalence of OTs was almost double in 2005–2016 (6.08%) as compared to overall prevalence over 30 year period (1990–2019); 3.92% and in comparison, to period before WHO 2005 (1990–2004); 3.87% and after WHO 2017 (2017–2019); 3.47% classification (Table 2, Table 3).

**Table 2:** Prevalence and frequency distribution of odontogenic tumors before WHO 2005 (1990–2004), after 2005 (2005–2016) and after 2017 (2017–2019) classification period.

S. no	Odontogenic tumors	1990–2004 (14 years)	2005–2016 (12 years)	2017–19 (3 years)
**A**	**Benign tumors**			
1	Ameloblastoma	71 (50.7%)	93 (38.11%)	34 (85%)
2	KCOT	0 (0.00%)	77 (31.56%)	0 (0.00%)
3	AOT	25 (17.86%)	19 (7.79%)	4 (10%)
4	Odontoma	22 (15.71%)	23 (9.43%)	0
5	Odontogenic fibroma	13 (9.29%)	1 (0.40%)	0 (0.00%)
6	CEOT	4 (2.86%)	5 (0.82%)	0 (0.00%)
7	Odontogenic myxoma	1 (0.71%)	4 (1.63%)	0 (0.00%)
8	CCOT	0 (0.00%)	4 (1.64%)	0 (0.00%)
9	Ameloblastic fibro-dentinoma, fibro-odontoma	3 (2.14%)	0 (0.0%)	0 (0.00%)
10	Cementoblastoma	0 (0.00%)	3 (1.23%)	0 (0.00%)
11	Ameloblastic fibroma	1 (0.71%)	1 (0.40%)	0 (0.00%)
12	DGCT	0 (0.00%)	2 (0.82%)	1 (2.5%)
13	SOT	0 (0.00%)	1 (0.40%)	0 (0.00%)
**B**	**Malignant tumors**			
14	Clear cell odontogenic carcinoma	0 (0.00%)	6 (2.46%)	0 (0.00%)
15	Ameloblastic carcinoma	0 (0.00%)	3 (1.23%)	1 (2.5%)
16	Malignant ameloblastoma	0 (0.00%)	2 (0.82%)	0 (0.00%)
	Total OT	140 (100%)	244 (100%)	40 (100%)
	Total biopsies	3622	4012	1153
	Prevalence	3.87%	6.08%	3.47%

KCOT, keratocystic odontogenic tumor; AOT, adenomatoid odontogenic tumor; CCOT, calcifying cystic odontogenic tumor; CEOT, calcifying epithelial odontogenic tumor (Pindborg’s tumor); DGCT, dentinogenic ghost cell odontogenic tumor; SOT, squamous odontogenic tumor; OT, odontogenic tumors.

**Table 3:** Prevalence, relative frequency, and gender distribution of Odontogenic tumors from 1990 to 2019 period after reinterpretation as WHO 2017 system of Odontogenic tumors’ classification.

S. no	Odontogenic tumors	Frequency distribution number (percentage)	Male	Female	*p*-Value
	Benign epithelial tumors				
1	Ameloblastoma	203 (58.84%)	105	98	0.273
2	AOT	48 (13.91%)	18	29	
3	CEOT	9 (2.60%)	4	3	
4	SOT	1 (0.28%)	0	1	
	Benign mixed epithelial and mesenchymal tumors				
5	Odontoma	46 (13.33%)	26	20	0.493
6	Ameloblastic fibroma	1 (0.28%)	0	1	
7	DGCT	3 (0.86%)	2	1	
	Benign mesenchymal tumors				
8	Odontogenic fibroma	15 (4.34%)	9	6	0.417
9	Odontogenic myxoma	5 (1.44%)	4	1	
10	Cementoblastoma	3 (0.86%)	1	2	
	Malignant tumors				
11	Clear cell odontogenic carcinoma	6 (1.73%)	4	2	0.376
12	Ameloblastic carcinoma	5 (1.44%)	2	3	
	Total	345 (100%)	175	167	0.522

AOT, adenomatoid odontogenic tumor; CEOT, calcifying epithelial odontogenic tumor (Pindborg’s tumor); DGCT, dentinogenic ghost cell odontogenic tumor; SOT, squamous odontogenic tumor; OTs, odontogenic tumors.

*Malignant ameloblastoma as it is included under ameloblastoma as per WHO 2017 classification, so we have included it under ameloblastoma only.

**Ameloblastic fibro-dentinoma and fibro-odontoma are not listed in WHO 2017 classification so are included under developing odontomas only.

***Total odontogenic tumors including calcifying cystic odontogenic tumor and keratocystic odontogenic tumor were 424. However, both of them are reconsidered as cysts in WHO 2017 system, thus we have excluded them from the total odontogenic tumor number (345 as compared to 424).

Relative frequency of OTs in three periods of classification, irrespective of time period, ameloblastoma (AME) remained the most common OT. During 1990–2004 period (WHO 1992 classification), adenomatoid odontogenic tumor (AOT: 17.86%) and odontoma (15.71%) followed AME. However, 2005–2016 period (WHO 2005 classification), keratocystic odontogenic tumor (KCOT: 31.56%) was second most frequent followed by odontoma (9.43%). During 2017–2019 (WHO 2017 classification), AOT (10%) ranked second after AME (Table 2).

Overall relative frequency of OTs: While reclassifying all OTs (1990–2019) as per 4^th^ edition of WHO classification; total 345 OTs were reported with prevalence of 3.92%. 96.81% tumors constituted benign and 3.81% malignant OTs. Benign epithelial tumors (Group 1a = 75.6%) were most predominant followed by benign mixed (Group 1b = 14.49%) (Table 3). The frequency distribution of histological subtypes of AME is illustrated in Table 4. The malignant tumors reported were clear cell odontogenic carcinoma (CCOC = 1.73%) and ameloblastic carcinoma (AC = 1.44%). Among variants of OTs, one case of clear cell variant of calcifying epithelial odontogenic tumor (CEOT) was reported.

**Table 4:** Distribution of ameloblastoma as per histological typing.

Histological subtypes of ameloblastoma	Number	Percentage	Mean age (years)
Unicystic	68	33.49%	32.47
Plexiform	46	22.60%	25.7
Follicular	33	16.20%	45.2
Acanthomatous	22	10.83%	36.36
Granular	14	6.89%	50.25
Desmoplastic	8	3.94%	51.88
Basal	2	0.98%	22.5
Malignant	2	0.98%	32
Unclassified	6	2.95%	–
Total	203	100%	

Gender distribution: Regarding gender distribution of 345 OTs, most of the benign OTs showed male predilection. However, AOT and cementoblastoma (CB); including isolated cases of squamous odontogenic tumor (SOT) and ameloblastic fibroma (AF) occurred in females. Among malignant OTs, CCO and AC showed male (2:1) and female (1:1.5) predominance respectively (Table 2, Table 3). The age of the patients at the time of diagnosis ranged from 6 to 75 years (mean = 31.9). The malignant OTs occurred in a relatively elder age group, while benign tumors which showed wide variation (Table 5).

**Table 5:** Age distribution of 345 odontogenic tumors.

Tumor	Total	Age at diagnosis (years)										
		Age										
		0–9	10–19	20–29	30–39	40–49	50–59	60–69	>70	Age range	Mean ± SD	Median
Benign epithelial tumors (1a)												
Ameloblastoma	203	–	36	46	38	38	28	8	9	11–75	35.16	32
AOT	48	1	25	15	4	1	–	1	–	6–61	20.4	18
CEOT	9	–	–	1	2	–	4	1	–	27–65	46.75	54.5
SOT	1			1							24	
Benign mixed epithelial and mesenchymal tumors (1b)												
Odontoma	46	3	24	12	1	3	4	–	–	6–55	22.73	19
Ameloblastic fibroma	1	–	1	–	–	–	–	–	–	14	14	
DGCT	3	–	–	–	–	1	1	1	–	44–61	53.3	55
Benign mesenchymal tumors [1c]												
Odontogenic fibroma	15	1	9	2	2	–	–	1	–	8–60	21.67	16
Odontogenic myxoma	5	–	1	3	1	–	–	–	–	16–30	23.8	30
Cementoblastoma	3	–	2	1	–	–	–	–	–	18–23	19.67	18
Malignant tumors												
Clear cell odontogenic carcinoma	6	–	–	–	2	2	–	2	–	37–65	49	45
Ameloblastic carcinoma	5	–	–	–	1	2	–	2	–	35–65	51	45
Total	345	6	97	81	51	46	36	15		6–75	31.9	30

AOT, adenomatoid odontogenic tumor; CEOT, calcifying epithelial odontogenic tumor (Pindborg’s tumor); SOT, squamous odontogenic tumor; DGCT, dentinogenic ghost cell odontogenic tumors; SD, standard deviation.

Site and region distribution: In this series of OTs, seven tumors were extra-osseous including three AOTs, one SOT and three odontogenic fibroma (OF). The mandible was the predominant site with maxilla to mandible ratio of 1:5.03. 7.14% of maxillary and 3.19% of mandibular tumors expanded and crossed the midline. Ramus involvement was seen in 102 (36.17%) mandibular tumors, however, data could not be retracted for sinus involvement in maxillary lesions (Table 6, Table 7). With regard to region distribution, most of the benign tumors predominantly occurred in mandibular posterior (premolar and molar) region. However, desmoplastic ameloblastoma (DA) and AF showed predominance in mandibular anterior; whereas AOT and isolated case of SOT in maxillary anterior region. With regard to malignant tumors, AC most frequently occurred in the mandibular posterior region, whereas CCOC showed equivocal distribution in mandibular anterior and posterior region.

**Table 6:** Site and region distribution of 345 Odontogenic tumors.

Tumor	Total	Mandible								Maxilla						NS	Maxilla: mandible ratio
		Anterior	Posterior (premolar and molar region)	Angle	Unilateral anterior to posterior	Crossing midline	Ramus involved	NS	Total	Anterior	Posterior	Unilateral anterior to posterior	Crossing midline	NS	Total		
																	
Benign epithelial tumors																	
Ameloblastoma	203	32	150	2	9	5	92 + 4^d^	1	193	4	4	–	–	–	8	2	1:24.1
AOT	48	7	8		1	4	–	–	2-	14^c^	6	4	3	–	27	1	1:1.35
CEOT	9	–	7	–	–	–	7 + 0^d^	–	7	–	–	–	–	–	–	2	
SOT	1^a^	–	–	–	–	–	–	–	–	1	–	–	–	–	1	–	
Benign mixed epithelial and mesenchymal tumors																	
Odontoma	46	5	26	–	–	–	–	–	31	14	–	–	–	–	14	1	1:2.2
Ameloblastic fibroma	1	1	–	–	–	–	–	–	1	–	–	–	–	–	–	–	
DGCT	3	1	2	–	–	–	–	–	3	–	–	–	–	–	–	–	
Benign mesenchymal tumors																	
Odontogenic fibroma	15	–	8^b^	–	2	–	1	–	11	–	1	1	1	–	3	1	1:3.67
Odontogenic myxoma	5	–	4	–	–	–	3 + 0^d^	–	4	–	1	–	–	–	1	–	4:1
Cementoblastoma	3	–	3	–	–	–	–	–	3	–	–	–	–	–	–	–	
Malignant tumors																	
Clear cell odontogenic carcinoma	6	2	2	–	–	–	–	–	4	1	1	–	–	–	2	–	1:2
Ameloblastic carcinoma	5	–	4	–	–	–	–	1	5	–	–	–	–	–	–	–	
Total	345	48	214	2	12	9	102 + 5^d^	2	282	34	13	5	4	–	56	7	1:5.03

AOT, adenomatoid odontogenic tumor; CEOT, calcifying epithelial odontogenic tumor (Pindborg’s tumor); DGCT, dentinogenic ghost cell odontogenic tumor; NS, not specified; SOT, squamous odontogenic tumor; OTs, odontogenic tumors.

a SOT was peripheral in location.

b Out of these odontogenic fibromas, 3 were peripheral in location.

c Out of these 14 AOTs, 3 AOT affecting maxillary anterior region were peripheral in location.

d Isolated ramus was involved in 5 cases but as an extension from posterior part of body of mandible to ramus was seen in 102 cases. First number indicates as a part of extension form posterior part of mandible and second number indicates isolated ramus involvement.

**Table 7:** Association between Site and frequency distribution of Odontogenic Tumors.

S. no	Odontogenic tumors	Frequency distribution	Mandible	Maxilla	Unspecified	*p*-Value
	Benign epithelial tumors					
1	Ameloblastoma	203 (58.84%)	193	8	2	0.001^a^ (s)
2	AOT	48 (13.91%)	20	27	1	
3	CEOT	9 (2.60%)	7	0	2	
4	SOT	1 (0.28%)	0	0	1	
	Benign mixed epithelial and mesenchymal tumors					
5	Odontoma	46 (13.33%)	31	14	1	0.761
6	Ameloblastic fibroma	1 (0.28%)	1	0	0	
7	DGCT	3 (0.86%)	3	0	0	
	Benign mesenchymal tumors					
8	Odontogenic fibroma	15 (4.34%)	11	3	1	0.851
9	Odontogenic myxoma	5 (1.44%)	4	1	0	
10	Cementoblastoma	3 (0.86%)	3	0	0	
	Malignant tumors					
11	Clear cell odontogenic carcinoma	6 (1.73%)	5	1		0.338
12	Ameloblastic carcinoma	5 (1.44%)	5	0		
	Total	345 (100)	283	45	4	0.001^a^(s)

AOT, adenomatoid odontogenic tumor; CEOT, calcifying epithelial odontogenic tumor (Pindborg’s tumor); DGCT, dentinogenic ghost cell odontogenic tumor; SOT, squamous odontogenic tumor.

## Discussion

The prevalence of OTs in 2005–2016 period increased approximately 57.10% (6.08%) when compared with 1990–2004 (3.87%) and 2017–2019 (3.47%) time period. This was in accordance with previous studies using WHO 2005 classification as compared to the second edition (1992).^30^, ^31^, ^32^, ^33^ The reason can be hypothesized to the attributed significant difference in prevalence owing to re-classification of OKC under neoplasm category (77 [31.56%] cases of KCOT in the present study) in 2005–2016 period; however, rarity of COC (4 cases, 1.64%) did not contribute much to increase in prevalence.

In this study, benign OTs were predominant, constituting 96.8% of all reported OTs following previous studies.^1^, ^3^, ^5^, ^30^^,^^32^, ^33^ The neoplasms of group 1a were most common as compared to 1b and 1c. This was in agreement with the previous studies^6^, ^34^ but in contrast to other studies which revealed 1b as a the most common group.^7^, ^15^ Almost all the OTs in the present study were diagnosed in patients of above 5 years (mean 31.9-years), strengthening the fact the OTs develop from remnants of tooth germ after crown completion.^1^

Regarding gender distribution, slight male predominance (1.04:1) in the present study was following previous study with male to female ratio of 1.2:1.^1^ Most of the studies in literature showed equal gender distribution in OTs but in contrast, female predominance is seen in few reports.^35^, ^36^

In our study, using either edition of WHO classification, AME constituted the commonest OT (Table 2). AOT was the second most common tumor in 1990–2004 and 2017–2019 periods whereas KCOT was the second most common tumor followed by odontoma in the 2005–2016 period. Similar to the present study, different studies have also reported variation in the frequency distribution of OTs after inclusion of KCOT. Few studies reported KCOT as the second most common tumor after AME similar to our study.^5^, ^8^, ^9^, ^16^^,^^18^, ^21^ However, other studies reported KCOT as the most common tumor followed by ameloblastoma or odontoma.^16^, ^19^, ^30^, ^31^, ^32^, ^33^, ^34^, ^36^, ^37^

While reclassifying all reported OTs according to WHO 2017 classification, overall, the most common tumor was AME followed by AOT and odontoma. This was similar to some studies from India, China, Hong Kong, Africa and Turkey,^1^, ^3^, ^6^, ^7^^,^^10^, ^15^, ^21^, ^34^^,^^35^, ^38^, ^39^, ^40^ and in contrast to other studies from Chile, Canada, Mexico, Sweden, Germany and U.S which showed odontomas as the most frequent OTs.^7^, ^11^, ^15^, ^30^^,^^31^, ^35^, ^41^, ^42^ The high frequency of AME in the Indian population may be because of its highly aggressive behavior causing incapacitating symptoms seeking medical consultation.

As odontomas are mostly asymptomatic, the reported lesser frequency than AME [including present study (13.33% vs. 58.84% of AME) may be explained by the fact that people usually do not seek medical advice unless severe symptoms suggesting an obvious pathology are present.^1^, ^7^ Secondly, it may suggest under-reporting because the diagnosis is usually made on radiographic presentation and specimens are not submitted to pathology.^1^, ^7^ Other reasons for low prevalence of odontoma at our centre might be because it is a tertiary care center and patients usually report with challenging pathologies similar to the observation by Gill et al.^18^ Also, it may reflect a true difference in the relative frequency of these tumors due to geographic variations.

AME showed a wide age range in the present series (11–75 years) with a mean of 35.16 years. Reichart et al. described 39.9 years age for developed countries in comparison to 27.7 years for developing countries for AME.^43^ The age in the present study (35.16) was intermediate between quoted values for industrialized and developing countries. Unicystic AME was the most dominant subtype (68 cases; 33.49%) followed by plexiform (22.60%) AME. The high frequency of unicystic AME was in agreement with the study by Luo et al. (31.17%) and comparable to other studies in USA (46%) and Estonia (31.6%).^4^, ^12^, ^37^ However, it was in contrast to other studies showing a low prevalence of unicystic variant.^43^, ^44^ All subtypes (except DA in anterior mandible) occurred predominantly in the posterior mandible, which was in agreement with literature findings.^1^, ^11^, ^18^, ^31^ Slight male predominance seen in our study was following few studies,^2^, ^3^, ^18^, ^37^ however in contrast to others showing female predilection^13^, ^19^, ^36^ or no gender predilection.^2^

AOT made up 13.91% of all OTs in the present study which is comparable to one Indian hospital study.^1^ However its relative frequency was higher as compared to most studies of Hong Kong, China, England, Mexico, most regions of India and Egypt (Table 1).^3^, ^8^, ^16^, ^18^^,^^19^, ^21^, ^35^, ^37^ It was the second most frequent tumor after AME in our study in conformity with a few studies in Nigeria and India^1^, ^14^ but in contrast to African studies, which reported myxoma commoner than AOT.^10^, ^45^, ^46^ The tumor predominantly involved maxillary anterior region of females (mean age of 20.4 years), consistent with previous reports.^8^, ^11^, ^45^ This was following currently the held opinion that AOT shows female preponderance and its anterior location makes it amenable for detection at a younger age.^1^, ^6^, ^8^, ^28^ Other studies by Ochsenius et al.^7^ and Arotiba et al.^10^ showed contrasting results for gender distribution.

In the present study, odontoma occurred at a younger age (mean = 20.4 years) consistent with studies.^1^, ^6^, ^8^, ^17^^,^^28^ Male patients were more commonly affected following published report,^1^, ^46^ but in contrast to studies with female predilection^6^, ^8^, ^9^ and with no gender predilection.^1^, ^14^, ^28^ In our series, odontoma occurred predominantly in the posterior mandibular (56.12%) followed by maxillary anterior region (30%) in consistent relation with previous reports.^1^, ^8^, ^24^, ^28^ Reichart et al. has shown complex odontoma to be common in the posterior mandible and compound odontoma in anterior maxilla.^1^, ^28^ However categorization into complex and compound was not done in our study.

Relative frequency of OF (4.34%) conformed with one institutional study in India (4.8%),^1^ Nigeria (5.3%, 4.50%,^14^, ^46^ Brazil (3.78%),^5^ Canada (4.85%)^15^ and Chile (5.5%);^7^ however higher in contrast to China (0.3%, 1.64)^9^, ^37^ and other regions of India (1.31%, 0.98%, 0.93%).^16^, ^19^, ^20^, ^22^^,^^23^ The present study showed male predilection in consistent with data from Nigeria,^14^ India,^1^, ^16^ whereas other studies reported equal gender distribution^7^, ^24^ and some with female predilection.^5^, ^15^, ^37^

CEOT in the present series accounted for about 2.60% of OTs. This represents the rarity of this tumor following other studies.^1^, ^5^, ^8^, ^11^^,^^37^ The tumor showed slight male predominance in conformity with Egyptian^20^ and Nigerian population^14^ (exclusive occurrence in males). They were exclusively diagnosed in the posterior mandible in present study in agreement with published reports which also showed mandibular predominance.^1^, ^8^, ^14^, ^28^ 3.81% OTs constituted malignant tumors similar to Brazil (3.84%)^5^ Nigeria (3.4%)^14^ but in contrast to other reports of India (1.2%, 1.0%, 5.5%, 0.98%),^1^, ^16^, ^19^, ^24^ USA (1.1%),^11^ Nigeria (5.2%),^46^ and China (5.96%).^37^

Thus, this study highlights the impact of changing classification systems on the prevalence of OTs, which can pave the way for future research opportunities. Further, multicentric studies are advocated to emphasize the fact that the increased prevalence does not reflect an actual increase in OTs, but due to reclassification will further provide clarification of misunderstanding especially in influencing oral public health preventive programs. Further, this evidence is important for future classifications of OTs.

## Conclusions

In conclusion, we observed a marked impact of KCOT inclusion under OTs leading to increased prevalence of OTs in the 2004–2016 period as compared to before 2005 and after 2017 period. The geographical similarities and variations in relative frequency of various histological types of odontogenic tumors may reflect heterogeneous populations with variable genetic and environmental factors. This however, warrants further investigations.

## Conflicts of interest

The authors declare no conflicts of interest.
